# *Fusobacterium nucleatum* infection leading to rare hepatorenal abscess: a case report

**DOI:** 10.3389/fmed.2025.1540430

**Published:** 2025-08-28

**Authors:** Lei Jin, Changkang Chen, Hong Zhao, Lei Chen, Xian Wang, Qin Wang, Ji Yuan, Jinjin Li, Shen Xu, Runzhen Zhang, Wenwen Chu, Naifang Ye, Guizhou Zou, Jun Ye

**Affiliations:** ^1^Department of Infectious Diseases, The Second Hospital of Anhui Medical University, Hefei, Anhui, China; ^2^Department of Radiology, The Second Hospital of Anhui Medical University, Hefei, Anhui, China; ^3^Department of Urology, The Second Hospital of Anhui Medical University, Hefei, Anhui, China; ^4^Department of Pathology, The Second Hospital of Anhui Medical University, Hefei, Anhui, China; ^5^Department of Clinical Laboratory, The Second Hospital of Anhui Medical University, Hefei, Anhui, China; ^6^Department of Stomatology, The Second Hospital of Anhui Medical University, Hefei, Anhui, China

**Keywords:** *Fusobacterium nucleatum*, hepatorenal abscess, metagenomic next-generation sequencing (mNGS), infectious disease diagnosis, oral cavity infection

## Abstract

*Fusobacterium nucleatum* is a gram-negative anaerobic bacterium commonly associated with periodontal disease. However, its role in extraoral infections, particularly in immunocompetent individuals, is increasingly recognized. We report a rare case of hepatorenal abscess caused by F. nucleatum in a previously healthy woman, initially suspected to have a malignant tumor based on PET-CT findings. Next-generation sequencing (NGS) of abscess aspirate confirmed the pathogen. The patient responded well to targeted antibiotic therapy. This case highlights the importance of considering anaerobic pathogens in deep-seated abscesses and the utility of NGS in achieving accurate microbial diagnosis.

## 1 Introduction

*Fusobacterium nucleatum* is a Gram-negative anaerobic bacterium that is commonly found as the normal flora in the oral cavity, gastrointestinal tract, and reproductive tract. It typically exists as a- commensal organism and sometimes acts as a pathogenic agent leading to infections (1). Although *F. nucleatum* infections are most frequently observed in the oral cavity and head and neck region, particularly in periodontal disease, there are also reports showing that *F. nucleatum* can cause lung abscess, brain abscess, and sepsis (1–4). However, the liver remains a common site for intra-abdominal abscess formation. This is attributed to its dual blood supply from the hepatic- artery, hepatic veins, and portal vein. Studies have shown that 48% of intra-abdominal abscesses occur in the liver (5). What is of particular scientific interest is that cases of disseminated abscesses caused by *F. nucleatum*, especially in the abdominal cavity, are extremely rare.

This case report describes a rare instance of a *F. nucleatum* infection leading to multiple abscesses in the hepatorenal region, and explores the clinical manifestations, diagnosis, and treatment of this case while analyzing the pathogenic mechanism of *F. nucleatum* infection based on relevant literature.

## 2 Case description

**FIGURE 1:**
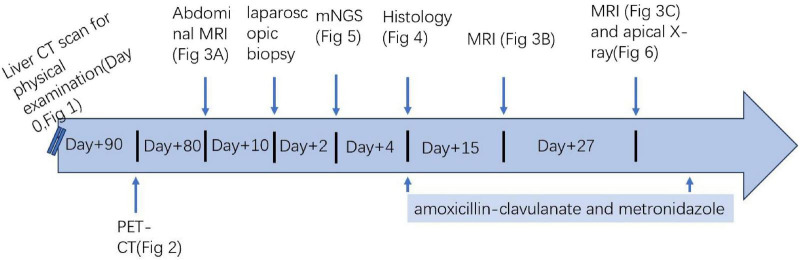
Summary of the patient’s clinical course, timeline of antibiotics administered. CT, computed tomography; PET-CT, positron emission tomography-computed tomography; MRI, magnetic resonance imaging; mNGS, metagenomic next-generation sequencing.

The patient is a 34 year-old female. Upon admission, through initial inquiry, she denied having a history of chronic illnesses such as diabetes, hypertension, or liver and kidney diseases. She had no known immunodeficiency, had never received immunosuppressive therapy, and had no history of non-steroidal anti-inflammatory drug (NSAIDs) use, nor a prior history of malignancy or recent infections. She denied smoking, alcohol abuse, or drug allergies with an unremarkable medical history. She was admitted to the Second Hospital of Anhui Medical University on August 1, 2024, because of the discovery of masses in the right kidney and right lobe of the liver during the past 2 months, as well as a lowgrade fever for 1 week. Prior to this, In February 2024, the patient had a chest CT scan at a local hospital, and the imaging of the liver showed a suspected low density lesion in the right lobe (Supplementary Figure 1). However, the patient had no discomfort such as fever, abdominal pain, or low back pain at that time, so further examinations were not performed. Subsequently, there were multiple space-occupying lesions in the liver, kidney, and the area between them during a physical examination in May 2024. The liver lesion’s diameter measured approximately 10 cm. At this time, the patient still had no obvious symptoms such as fever, abdominal pain, or back pain. She received further Positron Emission Tomography-Computed Tomography (PET-CT) scan at another hospital which also showed an irregular soft tissue shadow in the right kidney that was invading the adjacent right lobe of the liver and the space between the liver and kidney (see Figure 1 for the timeline). The boundaries between the liver parenchyma and the right adrenal gland were unclear, accompanied by multiple lymph node shadows and increased Fluorodeoxyglucose (FDG) metabolism. The result suggested malignant tumors, which were considered to be right renal malignancies showing liver and multiple lymph node metastases (Supplementary Figure 2). To assess the possibility of neoplasia in this lesion, the patient had liver, kidney, and perirenal tissue punctures at the external hospital. The pathology results indicated non-tumorous lesions, suggesting they were likely inflammatory conditions. The pathology slides and paraffin blocks of the lesions were sent to another hospital for consultation, and no clear evidence of malignancy was observed.

As previously mentioned, the patient was admitted to our hospital in August 2024, having experienced a week of fever prior to admission. Her body temperature usually does not exceed 38 degrees, with fatigue but no chills, poor appetite, abdominal pain, or bloating. At the time of admission, blood tests showed elevated white blood cells (WBC, 10.70 × 10^∧^9/L), with a neutrophil percentage of 76.0%, moderate anemia (hemoglobin 84 g/L), and a significantly elevated C-reactive protein (CRP) level (75.73 mg/L). The erythrocyte sedimentation rate (ESR) was 101 mm/h, indicating a marked increase. Procalcitonin, ferritin, and lactate dehydrogenase levels were within the normal reference ranges. Tests for anti-O, T-cell subsets, and immunoglobulins revealed no abnormalities, An abdominal MRI conducted on August 12, 2024, revealed multiple irregular patchy areas of hypointense signal on T1-weighted images and hyperintense signal on T2-weighted images in the right lobe of the liver, right kidney, and the interstitial region between them, with poorly defined and blurred lesion margins, suggesting inflammatory or infectious involvement (Supplementary Figure 3A). On August 22, 2024, laparoscopic biopsy of the tissue between the liver and kidney was performed. The postoperative pathology also revealed inflammatory lesions, showing no evidence of malignancy. The tissue histology and immunohistochemistry confirmed this finding later. As shown in Supplementary Figure 4, the lesion consisted mainly of blood clots, fibrin-like substances, and granulation tissue proliferation. At the same time, there was a significant infiltration of lymphocytes, plasma cells, neutrophils, and eosinophils, accompanied by pus-like material in some areas. The tissue sample from the area between the liver and kidney was sent for metagenomic next-generation sequencing (mNGS) simultaneously, which revealed an infection by *F. nucleatum* (Supplementary Figure 5 shows the result of a comparison between the detected *F. nucleatum* sequence and a reference sequence from the National Center for Biotechnology Information (NCBI) database).

To trace the pathway of bacterial invasion, we arranged a gastrointestinal endoscopy for the patient. Gastroscopy showed chronic gastritis with bile reflux, and colonoscopy revealed no abnormalities. After further detailed inquiry about the medical history, the patient recalled having undergone root canal treatment and restoration for her upper left second molar 2 years ago, though the specific details remained unclear. The filling material had later fallen out and was not replaced. Supplementary Figure 6 shows the patient’s periapical X-ray, which reveals that the crown of the left upper second molar is missing, and remnants of the root are still present. There are no filling materials visible in the root canal, and the distal alveolar bone is resorbed to half of the root. As an oral commensal bacterium, *F. nucleatum* might have invaded the body through the periapical tissues. Regarding antimicrobial treatment, we administered intravenous amoxicillin-clavulanate combined with metronidazole for 6 weeks. During this period, follow-up imaging showed gradual reduction of abscesses in the liver, kidney, and the hepatorenal space. After 6 weeks, the patient was switched to oral amoxicillin-clavulanate plus metronidazole for another 6 weeks, completing a total of 12 weeks of antibiotic therapy. During this period, her body temperature remained normal, and she experienced no other discomfort. Follow - up Magnetic Resonance Imaging (MRI) on September 14 and October 10, 2024, showed gradual shrinkage of the lesions in the liver, kidney, and between the liver and kidney (Supplementary Figures 3B, C). Although the sizes of the liver and kidneys were not measured before treatment, clinical signs and imaging findings indicated that the volumes of the liver and kidneys decreased after anti - infective treatment (Supplementary Figure 3D). During this period, C - reactive protein (CRP) and white blood cell count (WBC) returned to normal, and the erythrocyte sedimentation rate (ESR) showed a marked decrease. The patient is currently continuing oral anti - infective therapy.

## 3 Discussion

Common anaerobic bacteria which play significant roles in abdominal abscesses include *Bacteroides fragilis* and *Peptostreptococcus spp.* They usually result from dysbiosis of the intestinal flora, which can lead to complex infections and the formation of abscesses (6, 7). However, reports of *F. nucleatum* causing disseminated intra-abdominal abscesses, particularly in individuals without a history of surgery or trauma, are rare. Some studies indicate that the pathogenic mechanisms of *F. nucleatum* infections relate to its high invasiveness and adhesiveness, especially its ability to adhere to host cells and disrupt epithelial barrier function (2). The integrity of the epithelial barrier is crucial for defending against hematogenous dissemination of pathogens, analogous to the blood-brain barrier that protects the brain from toxins and pathogens. Existing studies have shown that outer membrane vesicles secreted by *F. nucleatum* may impair oral mucosal epithelial barrier function by inhibiting Claudin-4 expression (8). Concurrently, the bacterium can activate signaling pathways such as NF-κB through damaged barriers, inducing the expression of multiple cytokines and chemokines. Continuously released inflammatory factors disrupt the normal structure and function of the oral epithelial barrier, creating a mutually reinforcing cycle (9). This bacterium can traverse the damaged epithelial barrier and spread via the bloodstream from the oral cavity to distant organs. This route of dissemination may take place after periodontal infections or gingivitis, even when the patient exhibits no apparent oral symptoms (10). *F. nucleatum*, an opportunistic pathogen, typically invades during host immunocompromise. The patient underwent T-cell subset testing during hospitalization, which revealed normal immune function. A further review of the patient’s pre-illness status disclosed a history of oral diseases: root canal therapy and restoration were performed 2 years prior, and the endodontic filling material was not replaced after dislodgement. This suggests that oral pathologies and loss of root canal filling material provided preconditions for bacterial proliferation. Disruption of epithelial barrier function permitted bacteremia, leading to the development of intra-abdominal hepatorenal abscesses. Similarly, among 16 previously reported cases of pyogenic liver abscess caused by *F. nucleatum*, 5 patients had normal immune function (11). Notably, 4 of these 5 patients (80%) had dental diseases. Additionally, literature has shown that disseminated fusobacterial infections can occur in patients with oral diseases even in the absence of immunocompromise (10).

In this case, the patient did not exhibit significant signs of infectious toxicity during her illness, which is consistent with many reports on anaerobic infections (12, 13). The patient’s molar cavity and gingival inflammation served as a portal of entry. Hematogenous spread via the portal or systemic circulation may have seeded distant organs such as the liver and kidney. Anaerobic infections often present subtly, with systemic symptoms typically mild and routine infection indicators (such as leukocyte count or CRP) showing only slight elevations, which complicates clinical diagnosis. Additionally, the slow growth and oxygen sensitivity of these organisms often result in false-negative cultures, further challenging the identification of the causative pathogen. The patient sought medical attention because of multiple occupying lesions in the liver, kidney, and the area between the liver and kidney. PET-CT results suggested malignancy. However, biopsy results did not support a neoplastic process. This inconsistency between imaging and pathology suggests that clinicians should focus on other potential causes, especially the possibility of infectious diseases. Despite repeated attempts to culture the organism, no clear pathogen was identified, likely due to issues related to sample handling and culture methods. Studies show that anaerobic bacteria have poor cultivation sensitivity and high false-negative rates, which makes the identification of pathogens through traditional culturing methods challenging (14). In contrast, m-NGS has demonstrated clear advantages. It can identify a variety of pathogens by detecting the genomic sequences of microbial pathogens. This capability allows for the recognition of different pathogens without the need for cultivation, especially those that are difficult to cultivate or are rare. This technology relies on high-throughput sequencing of all microbial Deoxyribonucleic Acid (DNA) in a sample to analyze genetic information. This process enables rapid and accurate pathogen identification and opens new avenues for diagnosing infectious diseases (15). Thus in recent years, mNGS has gradually become an effective tool for diagnosing complex infections in clinical practice.

In terms of treatment, While *F. nucleatum* is generally susceptible to penicillin, increasing resistance has been reported. In a study, β-lactamase-producing *F. nucleatum* was detected in 72% of patients with refractory marginal periodontitis, yet remained susceptible to β-lactam combination formulations containing clavulanic acid (e.g., amoxicillin/clavulanic acid) (16). The 2019 Infectious Diseases Society of America (IDSA) guidelines recommend β-lactam/β-lactamase inhibitor combinations as first-line therapy for suspected β-lactamase-producing anaerobic infections (e.g., abdominal and oral-source abscesses). Additionally, a recent retrospective study in Japan (Asia-Pacific region) demonstrated that all isolated *F. nucleatum* strains were sensitive to β-lactam/β-lactamase inhibitors (17). Based on these findings and relevant guideline recommendations, we initiated antimicrobial therapy with amoxicillin-clavulanate and metronidazole for this patient. In this case, the patient demonstrated a favorable response to β-lactam/β-lactamase inhibitor combination therapy. This positive outcome further highlights the necessity of performing mNGS testing to identify pathogens for treatment guidance when feasible. Another important point is the role of epithelial and mucosal barriers in preventing microbial translocation. Disruption of these barriers, such as through dental procedures or poor oral hygiene, has been linked to systemic infections by oral pathogens (18). In this context, the molar lesion in our patient likely played a critical role in the pathogenesis.

This case also underscores the importance of clinical suspicion and diagnostic persistence. While FDG-PET is useful for identifying metabolically active lesions, it lacks specificity and can mislead clinicians toward a neoplastic diagnosis. Early microbiological investigation, especially using advanced tools like mNGS, can prevent misdiagnosis and guide timely treatment.

## 4 Conclusion

This case demonstrates a rare instance of hepatorenal abscesses caused by *F. nucleatum*. During the diagnostic workup, repeated pathological examinations and pathogen cultures failed to identify the etiology, highlighting the limitations of traditional tests in infections caused by uncommon pathogens. By detecting all microbial nucleic acids in the specimens, mNGS (metagenomic next-generation sequencing) rapidly identified *F. nucleatum*–a pathogen often overlooked by conventional methods–in this case, providing critical evidence for precision antimicrobial therapy. Although mNGS is costly, its potential benefits in reducing invasive procedures, avoiding unnecessary surgeries, and shortening hospital stays justify the balance of risks and benefits. Clinicians should proactively integrate mNGS when traditional approaches are inadequate to enhance diagnostic efficiency and improve patient outcomes.

## Data Availability

The original contributions presented in this study are included in this article/Supplementary material, further inquiries can be directed to the corresponding author.

## Supplementary material

Supplementary Figure 1 Liver CT results of the patient from an external hospital in February 2024. CT, computed tomography.

Supplementary Figure 2 PET-CT results of the patient from an external hospital on May 22, 2024. PET-CT, Positron Emission Tomography-Computed Tomography.

Supplementary Figure 3 Dynamic review of the patient’s abdominal MRI during anti-infective treatment [**(A)** Axial abdominal MRI images from August 12, 2024, show the liver, the region between the liver and kidney, and the kidney area. **(B)** Axial abdominal MRI images from September 13, 2024. **(C)** Axial abdominal MRI images from October 10, 2024. **(D)** Coronal plane abdominal MRI on August 12, 2024, September 13, 2024, and October 10, 2024). MRI, magnetic resonance imaging.

Supplementary Figure 4 Pathological biopsy of the mass between the patient’s liver and kidney showed that the lesion mainly consisted of blood clots, fibrin-like material, and granulation tissue proliferation, with abundant infiltration of lymphocytes, plasma cells, neutrophils, and eosinophils. Some areas showed pus-like material [see **(A)** at 100X, **(B)** at 400X; blue–neutrophils, red–plasma cells, green—lymphocytes, yellow–eosinophils].

Supplementary Figure 5 The patient’s *Fusobacterium nucleatum* sequence comparison with a reference sequence from the NCBI database. NCBI, National Center for Biotechnology Information.

Supplementary Figure 6 The patient’s apical X-ray.
