# Supplementary figures and images for: Fusobacterium nucleatum infection leading to rare hepatorenal abscess: a case report
Source: Front Med (Lausanne). 2025 Aug 28;12:1540430. doi: 10.3389/fmed.2025.1540430 (PMC12424139; doi:10.3389/fmed.2025.1540430)

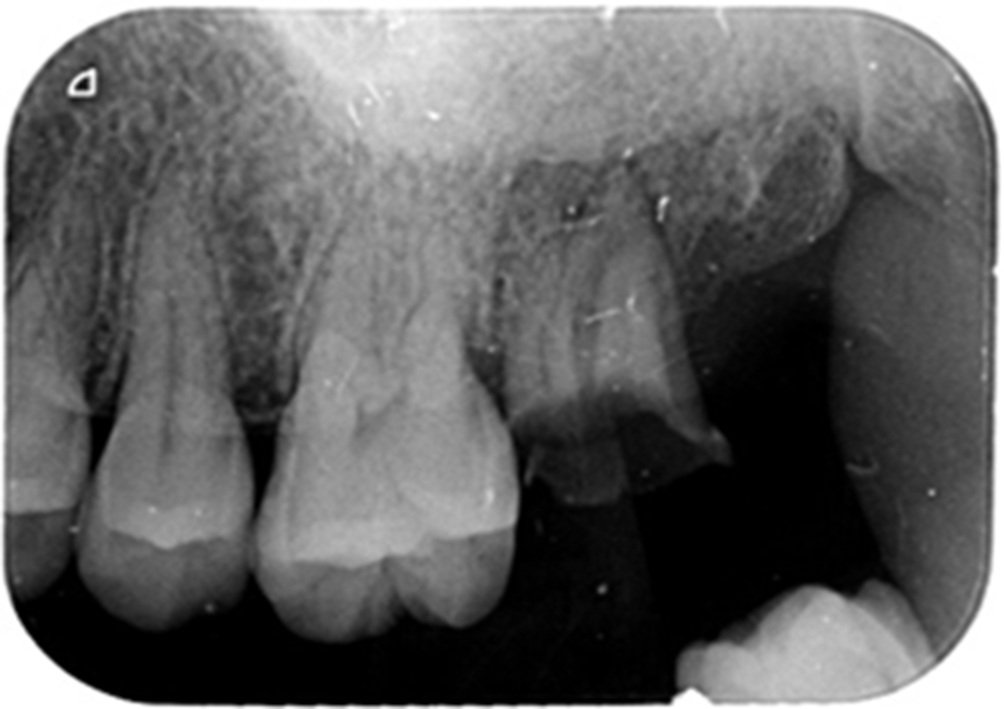

Supplement: Supplementary Figure 1 — Liver CT results of the patient from an external hospital in February 2024. CT, computed tomography. [file Supplementary_file_1.zip › 1540430-Supplementary Data-Figure 6/Image 9.JPEG]

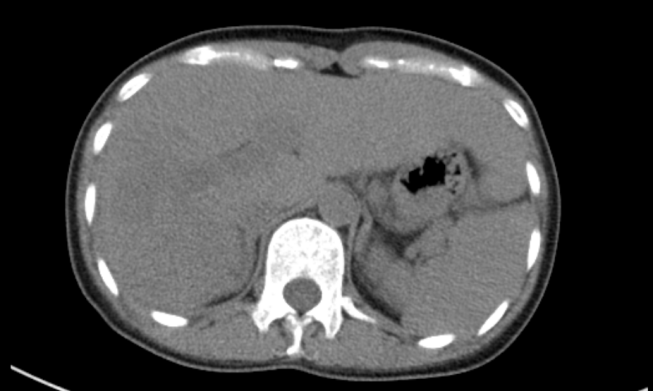

Supplement: Supplementary Figure 1 — Liver CT results of the patient from an external hospital in February 2024. CT, computed tomography. [file Supplementary_file_1.zip › 1540430-Supplementary Data-Figure 1/Image 10.PNG]

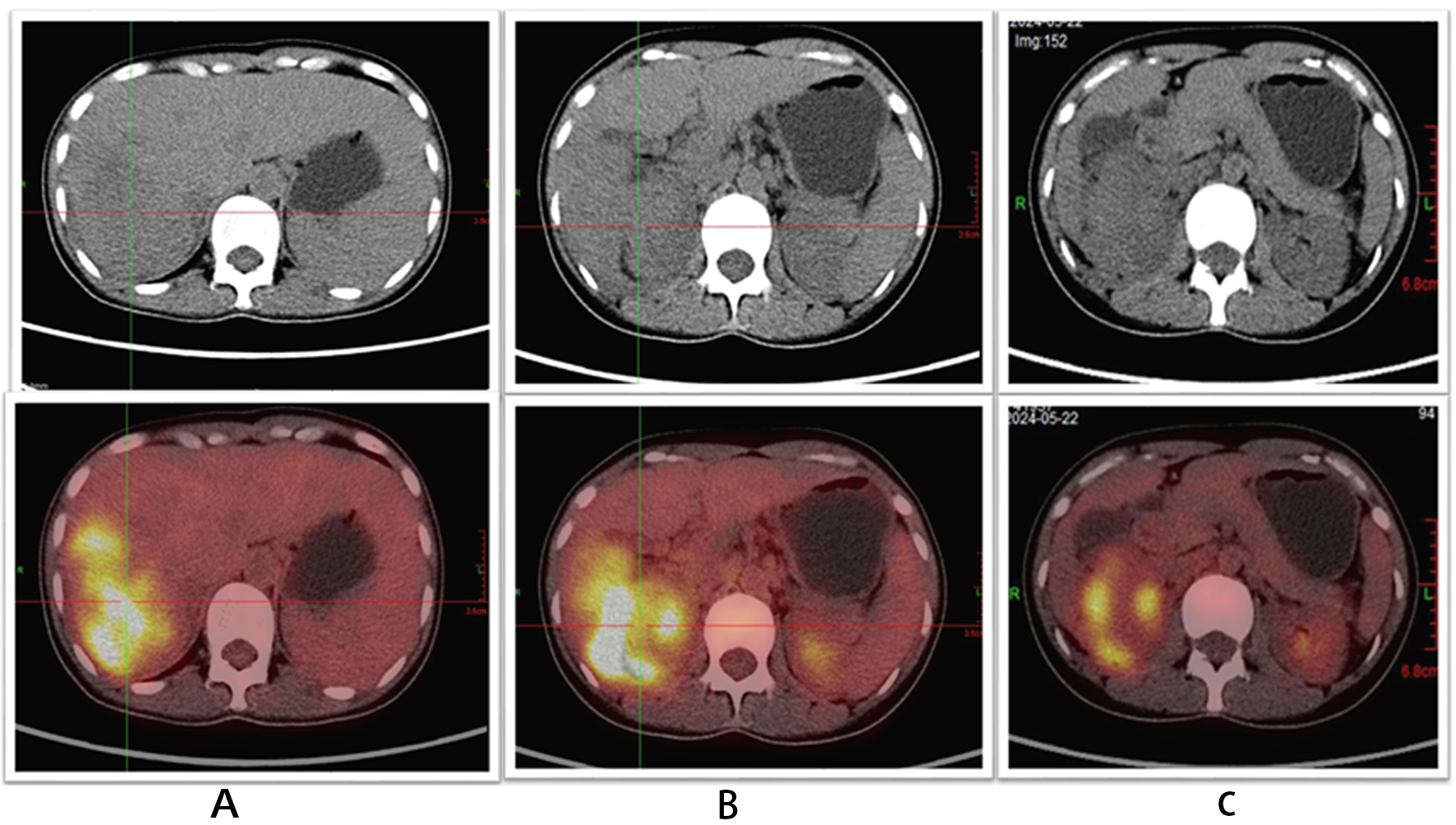

Supplement: Supplementary Figure 1 — Liver CT results of the patient from an external hospital in February 2024. CT, computed tomography. [file Supplementary_file_1.zip › 1540430-Supplementary Data-Figure 2/Image 1.JPEG]

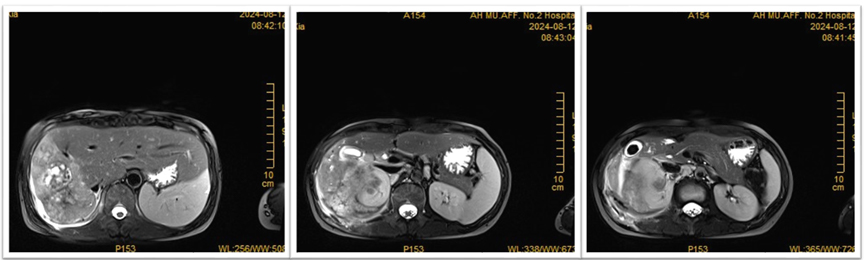

Supplement: Supplementary Figure 1 — Liver CT results of the patient from an external hospital in February 2024. CT, computed tomography. [file Supplementary_file_1.zip › 1540430-Supplementary Data-Figure 3/A.JPEG]

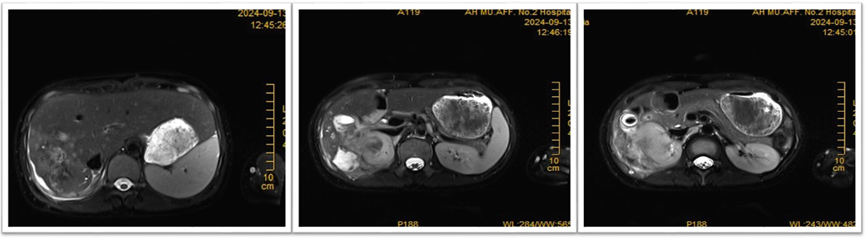

Supplement: Supplementary Figure 1 — Liver CT results of the patient from an external hospital in February 2024. CT, computed tomography. [file Supplementary_file_1.zip › 1540430-Supplementary Data-Figure 3/B.JPEG]

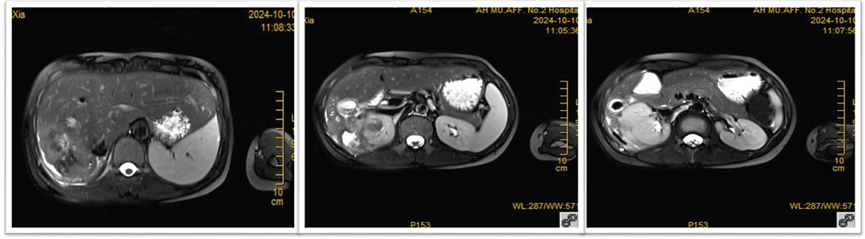

Supplement: Supplementary Figure 1 — Liver CT results of the patient from an external hospital in February 2024. CT, computed tomography. [file Supplementary_file_1.zip › 1540430-Supplementary Data-Figure 3/C.JPEG]

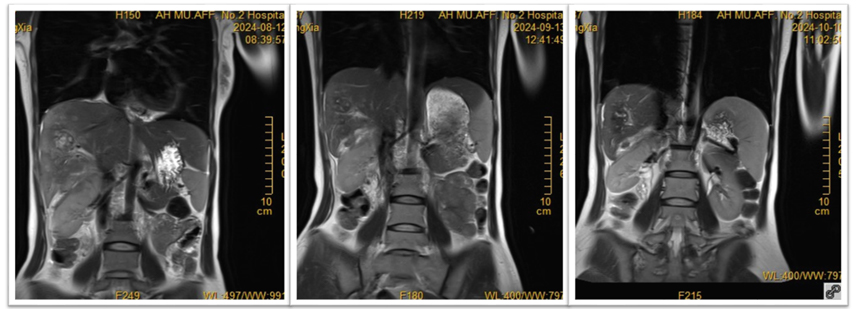

Supplement: Supplementary Figure 1 — Liver CT results of the patient from an external hospital in February 2024. CT, computed tomography. [file Supplementary_file_1.zip › 1540430-Supplementary Data-Figure 3/D.JPEG]

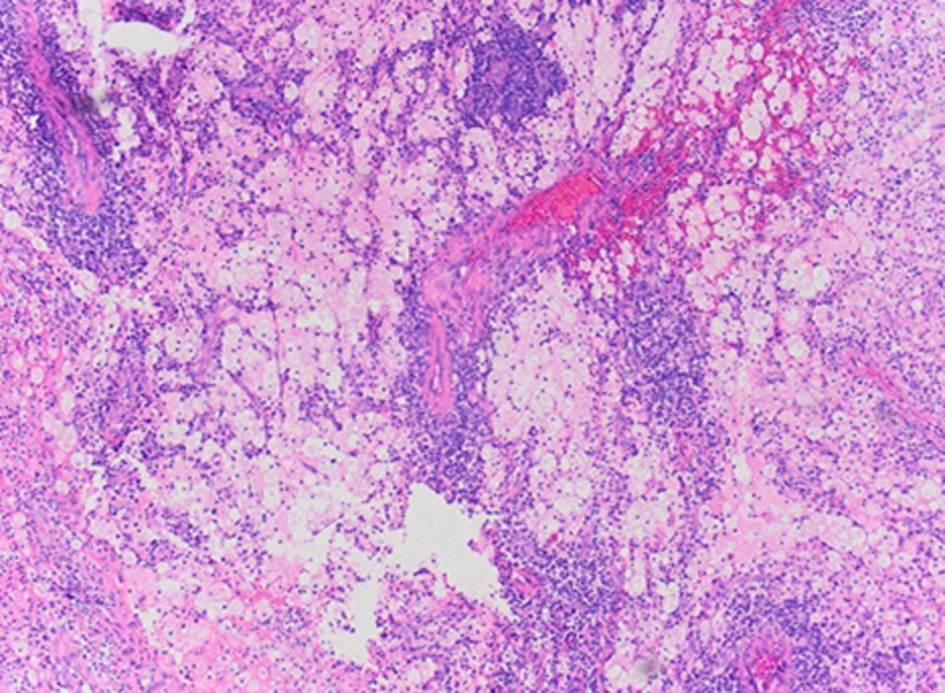

Supplement: Supplementary Figure 1 — Liver CT results of the patient from an external hospital in February 2024. CT, computed tomography. [file Supplementary_file_1.zip › 1540430-Supplementary Data-Figure 4/A.JPEG]

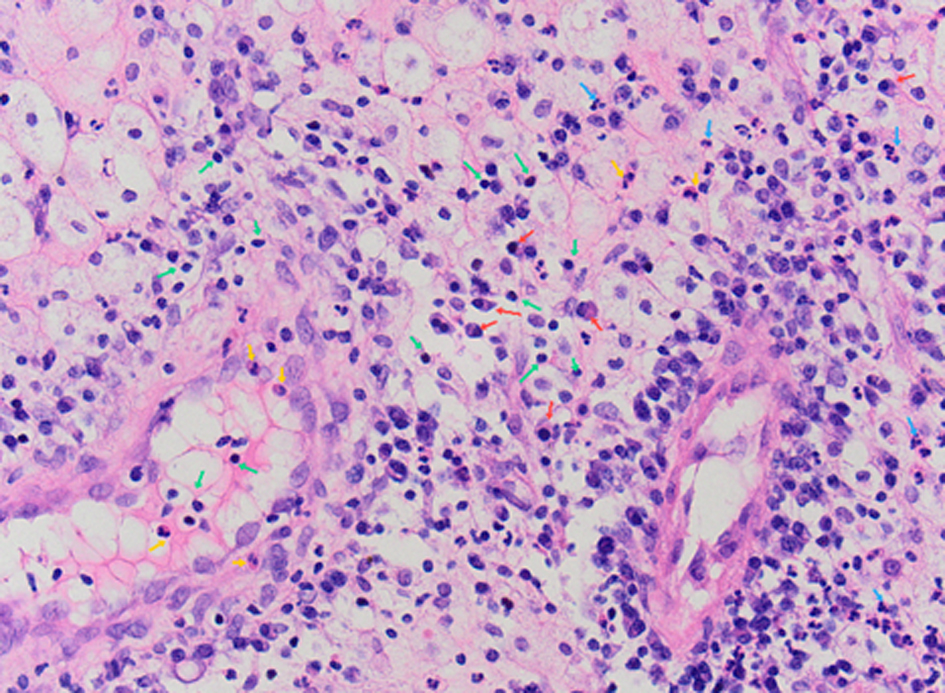

Supplement: Supplementary Figure 1 — Liver CT results of the patient from an external hospital in February 2024. CT, computed tomography. [file Supplementary_file_1.zip › 1540430-Supplementary Data-Figure 4/B.JPEG]

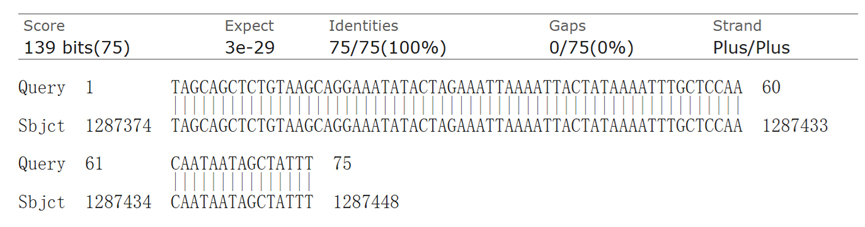

Supplement: Supplementary Figure 1 — Liver CT results of the patient from an external hospital in February 2024. CT, computed tomography. [file Supplementary_file_1.zip › 1540430-Supplementary Data-Figure 5/Image 8.JPEG]
